# Fishborne Zoonotic Intestinal Trematodes, Vietnam

**DOI:** 10.3201/eid1312.070554

**Published:** 2007-12

**Authors:** Do Trung Dung, Nguyen Van De, Jitra Waikagul, Anders Dalsgaard, Jong-Yil Chai, Woon-Mok Sohn, K. Darwin Murrell

**Affiliations:** * National Institute of Malariology, Parasitology and Entomology, Hanoi, Vietnam; † Mahidol University, Bangkok, Thailand; ‡ Hanoi Medical University, Hanoi, Vietnam; § University of Copenhagen, Frederiksberg, Denmark; ¶ Seoul National University College of Medicine, Seoul, South Korea; # Gyeonsang National University, Jinju, South Korea

**Keywords:** Zoonosis, intestinal trematodes, fishborne parasites, aquaculture, public health, hookworms, whipworms, roundworms, Vietnam, research

## Abstract

Although fishborne zoonotic trematodes that infect the liver are well documented in Vietnam, intestinal fishborne zoonotic trematodes are unreported. Recent discoveries of the metacercarial stage of these flukes in wild and farmed fish prompted an assessment of their risk to a community that eats raw fish. A fecal survey of 615 persons showed a trematode egg prevalence of 64.9%. Infected persons were treated to expel liver and intestinal parasites for specific identification. The liver trematode *Clonorchis sinensis* was recovered from 51.5%, but >1 of 4 intestinal species of the family Heterophyidae was recovered from 100%. The most numerous were *Haplorchis* spp. (90.4% of all worms recovered). These results demonstrate that fishborne intestinal parasites are an unrecognized food safety risk in a country whose people have a strong tradition of eating raw fish.

## Abstract 2

These parasites are an unrecognized food safety risk in a population with a tradition of eating raw fish.

Foodborne parasites are widespread and more common than generally recognized. Among these parasites, fishborne zoonotic trematodes (FZTs) are estimated to infect >18 million persons; worldwide the number at risk may be much greater (*1*–*3*). The FZTs include many species, especially representatives of the families Heterophyidae, Echinostomatidae, and Opisthorchiidae. Although their metacercarial cysts are easily inactivated by heating at 60°C or freezing to –20°C, they are highly prevalent in many regions, especially in Asia where food traditions include eating raw or improperly cooked fish dishes (Figure 1) (*4*). The fishborne liver flukes *Clonorchis sinensis*, *Opisthorchis viverrini*, and *O*. *felineus* cause cholangitis, pancreatitis, and cholangiocarcinoma in humans (*4*–*6*). During the past 10–20 years, a second large group of FZTs, the so-called minute intestinal flukes, has been increasingly recognized as widely distributed and a cause of illness (*1*–*8*).

**Figure 1:**
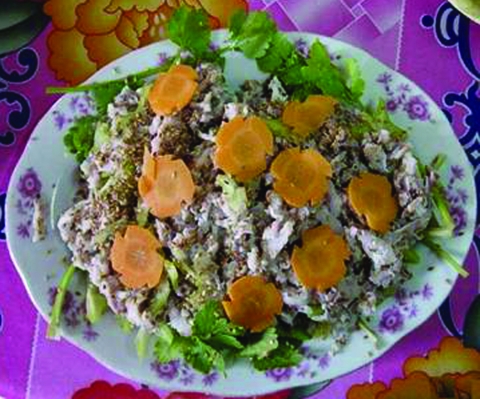
Typical dish of raw fish (slices of silver carp) sold in Vietnamese restaurants.

The exponential increase in aquaculture is suggested to be the major cause of the emergence of FZTs in east and Southeast Asia (*2*,*3*). For example, in the People’s Republic of China, the land devoted to aquaculture increased 75% (to 4.9 million hectares) since 1970, accompanied by a tripling of cases of infection with *C*. *sinensis* (*3*). The association of *O*. *viverrini* in Thailand and Lao People’s Democratic Republic with fisheries has also been reported (*9*,*10*). However, wild fish are also frequently infected, but epidemiologic information to compare relative infection risks from eating wild fish and farmed fish in many FZT-endemic loci is insufficient (*1*,*4*).

A recent review of publications on FZTs in Vietnam indicated infections with only liver flukes (*C*. *sinensis* and *O*. *viverrini*) in humans (*11*). However, recent Vietnamese surveys for zoonotic parasites in cultured and wild fish in northern and southern Vietnam identified metacercarial stages of several zoonotic intestinal trematode species in fish (*12*,*13*). This finding is of concern because fish production has increased 9.3-fold (to 400,000 tons) over the past 40 years in Vietnam (*3*). Furthermore, human intestinal flukes are highly prevalent in neighboring countries such as Thailand (*14*), Lao People’s Democratic Republic (*15*), and the People’s Republic of China (*16*), which further raises the issue of whether human infections might be present in Vietnam but overlooked because of diagnostic difficulties in differentiating liver and intestinal fluke eggs in fecal examinations (*11*,*17*). A more reliable approach to detect and characterize human FZTs is to treat egg-positive patients and recover and identify the expelled adult worms (*7*,*15*).

We conducted a study in April 2005 in Nam Dinh Province, an area of Vietnam in which persons are known for eating raw fish, and where previous investigations have shown a high prevalence of liver flukes (*11*,*13*). Identification of worms expelled from egg-positive persons showed that intestinal FZTs are present in Vietnam and represent a major public health risk for a population with the habit of eating raw fish.

## Materials and Methods

### Site, Sampling, and Examination Procedure

**Figure 2:**
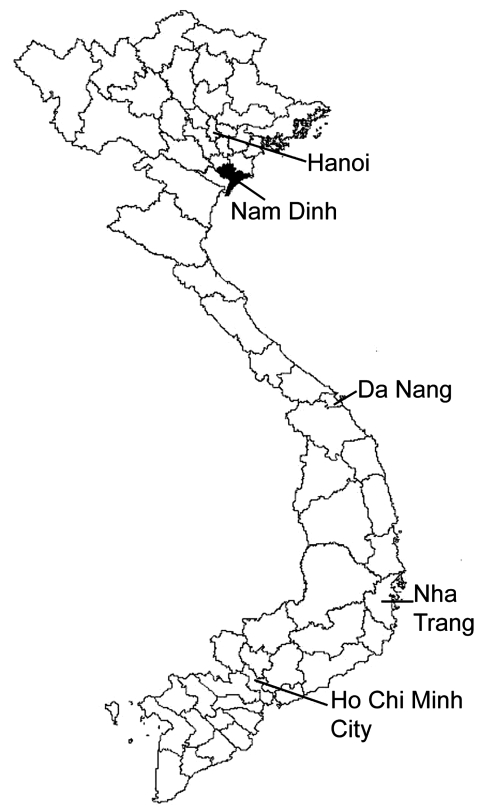
Map of Vietnam showing location of Nam Dinh Province, investigated for fishborne zoonotic trematode infections, April 2005.

A cross-sectional survey for fecal eggs was conducted in 2 communes in Nghia Hung District, Nam Dinh Province, Vietnam, southeast of the capital of Hanoi (Figure 2), a clonorchiasis-endemic area (*11*). The rural population in these communes is mostly farmers with fish ponds that are integrated into their farming systems, e.g., pig farming. In 2005, the Nghia Phu commune had a population of 9,608, including 2,214 families, and the Nghia Lac commune had a population of 9,147, including 2,160 families. Households in these 2 communes were randomly selected from a list provided by community authorities, and from each household 1 man or 1 woman who was head of household was selected. Trained personnel delivered labeled plastic containers to the selected persons and instructed them how to collect a fecal sample and store it until it was retrieved the next day. The label requested the person’s name, age, address, and date of stool collection.

Permission to conduct this research was obtained from the National Institute of Malariology, Parasitology and Entomology (NIMPE), Hanoi, and the Faculty of Tropical Medicine, Mahidol University, Bangkok. Each study participant signed a consent form, which is on file at NIMPE.

From each stool sample, 2 Kato-Katz smears were prepared and analyzed by using the standard kit provided to NIMPE by the World Health Organization and originally obtained from Vestegaard Frandsen Pvt. Ltd. (New Delhi, India). Fecal slides were examined by light microscopy (×400). Helminth eggs were identified and enumerated, and the number of eggs was multiplied by 23 to obtain the number of eggs per gram (epg) of feces.

### Parasite Expulsion

Thirty-three persons who had >1,000 epg on fecal examinations were selected for worm expulsion. Selected patients were asked to eat a light liquid dinner the evening before treatment. The following morning they were given oral praziquantel, 25 mg/kg; 1 hour later, they were given a saturated solution of 30 g of MgSO_4_ dissolved in water. Subsequently, 3–4 consecutive posttreatment stools were collected. Worms were recovered by a series of washing steps (*15*).

All persons who were positive for eggs were provided free drug treatment. Patients with nematode infections were given 1 dose of albendazole (400 mg) or mebendazole (500 mg), and patients with trematode infections were given praziquantel (25 mg/kg, 3× a day for 1 day).

### Identification of Adult Worms Recovered

Adult worms were identified by direct light microscopy while still alive; those that needed further examination were fixed in 10% formalin and stained with Semichon acetocarmine, mounted on a slide, and measured (*18*). Identifications were made by using published taxonomic references (*19*,*20*).

### Data Analysis

Results of fecal examinations for helminth eggs were analyzed for prevalence and intensity of infection (epg), as measured by enumeration of eggs per gram of feces. Species infection rates (number of expelled worms) were descriptively analyzed by using SPSS version 11.0 software (SPSS Inc., Chicago, IL, USA) and χ^2^ and Student *t* tests.

## Results

### Parasite Diversity, Prevalence, and Intensity

A total of 615 persons, 563 men (91.5%) and 52 women (8.5%), were selected and submitted stool for examination. Fecal egg examinations showed that 554 persons (90.1%) were positive for helminth parasites (Table 1). *Trichuris trichiura* (whipworm) nematode eggs were found in 58.2% of the stool samples. A total of 64.9% were infected with small trematode eggs (<50 μm long), and 39.5% were infected with *Ascaris lumbricoides* (roundworm). Hookworm eggs (3.1%) and large (>50 μm long) trematode eggs (0.8%) were infrequently seen (Table 1). Multiparasitism was common in this community, with 65.1% of the persons expelling eggs having >2 species or types of eggs. Small trematode eggs, all <50 μm long, were presumed to be either those of *C*. *sinensis* or of intestinal trematodes of the family Heterophyidae. However, differentiation was not considered reliable by light microscopy, and selected persons were treated to expel their helminth parasites.

**Table 1:** Helminth infections in persons living in Nghia Phu and Nghia Lac communes, Nam Dinh Province, Vietnam, April 2005

Helminth egg species or type	Fecal examination result, no. positive (%)
Small trematodes (<50 μm long)	399 (64.9)
Large trematodes (>50 μm long)	5 (0.8)
*Ascaris lumbricoides*	243 (39.5)
*Trichuris trichiura*	358 (58.2)
Hookworm	19 (3.1)
Total positive	554/615 (90.1)

On the basis of egg count data, small trematode infection prevalence differed significantly between men (68.7%) and women (23.1%) (χ^2^ 43.56, p<0.05). The infection rate for small trematode infection in men also differed significantly between age groups; it was significantly higher for those >40 years of age (χ^2^ 7.95, p<0.05). In contrast, women did not show a significant difference in infection rates between age groups (χ^2^ 0.85, p>0.05).

Most persons with small trematode eggs showed low infection intensity (epg); 344 (86.2%) of 399 shed <1,000 epg, and 55 (13.8%) of 399 shed 1,000–9,999 epg. Infection intensity differed significantly between those <40 years of age and those >40 years of age (χ^2^ 4.17, p<0.05) (Table 2).

**Table 2:** Intensity of small trematode infections in 2 age groups, Nam Dinh Province, Vietnam, April 2005*

Age group	No. positive	No. (%) with light infection†	No. (%) with moderate infection†
<40 y	111	102	9
>40 y	288	242	46
Total	399	344 (86.2)	55 (13.8)

*epg, eggs per gram (of feces). †Light infection = 1–999 epg; moderate infection = 1,000–9,999 epg.

The prevalence of *A*. *lumbricoides* was 39.3% in men and 42.3% in women. There was a significant increase in prevalence with age only in women (χ^2^ 6.4, p<0.05). Infection with *T*. *trichiura* infection did not differ significantly by sex or age (p>0.05).

### FZT Species Identification

**Figure 3:**
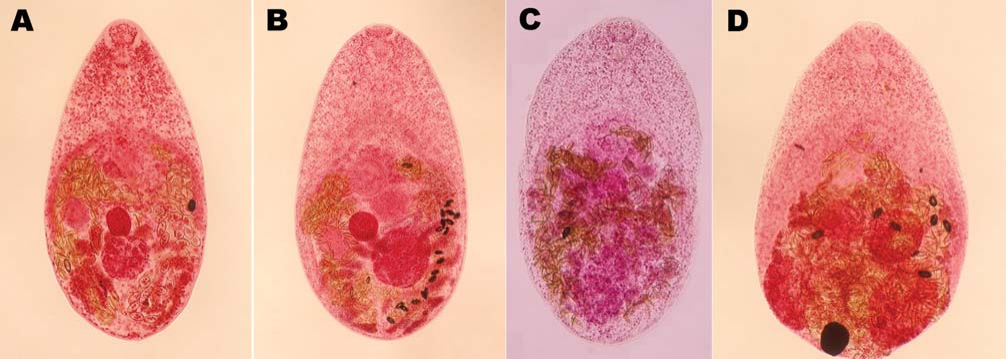
Adult trematodes isolated from Vietnamese persons. A) *Haplorchis pumilio*. B) *H. taichui*. C) *H. yokogawai*. D) *Stellantchasmus falcatus*. (Semichon acetocarmine stained, magnification ×120.)

Trematodes responsible for releasing small eggs were identified by using morphologic characterization of adult stages expelled from patients. A total 15,185 adult worms were collected from 33 patients. The number and prevalence of individual species of expelled trematodes are shown in Table 3. *C*. *sinensis* and 4 species of intestinal fishborne zoonotic flukes were identified (Figure 3); *C*. *sinensis* was isolated from 51.5% of patients. Intestinal fluke species identified (mean body length × width measurements in µm) were *Haplorchis pumilio* (632 × 291), *H*. *taichui* (756 × 421), *H*. *yokogawai* (760 × 400), and *Stellantchasmus falcatus* (468 × 298). Prevalence of intestinal flukes was *H*. *pumilio*, 100%; *H*. *taichui*, 69.7%; *H*. *yokogawai*, 6.1%; and *S*. *falcatus*, 3.0%. *H*. *pumilio* was the most common trematode (90.4%) of all worms isolated; 13,734 adult worms were isolated from 33 persons (mean 416.2); 1 patient expelled 4,525 worms. The plantborne intestinal pig trematode *Fasciolopsis buski* was isolated from 1 patient.

**Table 3:** Species and no. trematode adult worms recovered, Vietnam, Nam Dinh Province, April 2005

Trematode sp.	No. positive persons (%)	Total worms recovered	Average no. worms recovered (range)
Liver fluke			
*Clonorchis sinensis*	17 (51.5)	72	4.2 (1–18)
Intestinal flukes			
*Haplorchis pumilio*	33 (100)	13,734	416.2 (1–4,525)
*H. taichui*	23 (69.7)	1,323	40.1 (1–307)
*H. yokogawai*	1 (3.0)	3	3
*Stellantchasmus falcatus*	2 (6.1)	52	26 (15–37)
*Fasciolopsis buski*	1 (3.0)	1	1

**Figure 4:**
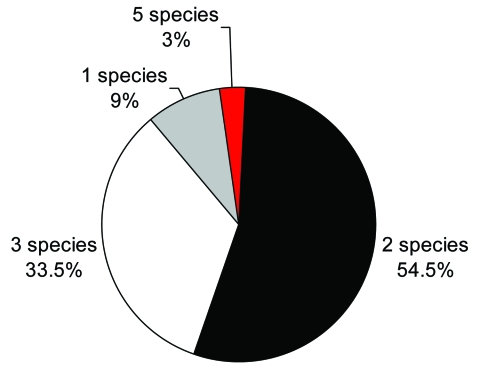
Multiple fishborne trematode infections in humans, Nam Dinh Province, Vietnam, April 2005.

Multiple infections with FZTs were common (Figure 4): 54.5% of patients were infected with 2 trematode species, 33.3% with 3 species. A total of 9% were infected with only 1 species. One person (3%) was infected with 4 FZT species and *F*. *buski*.

## Discussion

Our results demonstrate that zoonotic fishborne intestinal trematodes are endemic in Vietnam. These trematodes represent, to our knowledge, a new and previously unrecognized public health problem. To our knowledge, in the many publications on human parasites originating in Vietnam since the 19th century colonial era, no reports on these intestinal fishborne parasites have appeared (*11*). Whether this zoonosis is newly emerging in Vietnam because of changes in agriculture/aquaculture, demographics, social, or environmental changes or if it has been overlooked because of diagnostic problems is not known. However, snail vectors (e.g., *Melanoides tuberculata*) and suitable vertebrate intermediate (fish) and reservoir hosts (fish-eating birds, dogs, cats, pigs) for FZTs are common in this country (*1*,*4*,*11*,*19*,*21*). Furthermore, *H*. *taichui*, *H*. *pumilio*, *H*. *yokogawai*, *and S*. *falcatus* are endemic in neighboring countries such as Thailand (*14*), Lao People’s Democratic Republic (*10*,*15*), and the People’s Republic of China (*16*).

It is puzzling why zoonotic heterophyids have only recently been isolated from fish in Vietnam (*12*,*13*,*22*) if they are endemic. These parasites may have been recently introduced into this country and then became a zoonotic risk. Intensification of aquaculture, use of human and animal manure for pond fertilization, and increased consumption of fish because of increasing affluence by a population with a tradition of eating raw fish may be contributing factors for infection. These issues need to be investigated if effective means for prevention of transmission are to be developed. Use of manure and waste water in aquaculture is a well-recognized risk factor for trematode infections in fish (*2*,*12*,*13*) and has been the focus of 2 hazard analysis, critical control point–based control projects (*4*). Major sources of infected fish responsible for trematode transmission to humans must be ascertained because FZT metacercariae have been found in both wild and farmed fish in Vietnam, as well as elsewhere in Asia (*4*,*10*,*12*–*16*).

Public health and agricultural/fishery agencies should consider intestinal and liver flukes as an FZT complex because they share most biologic features and are risk factors for human infection. Although intestinal flukes are less well characterized clinically than liver flukes, they are increasingly being recognized as a cause of intestine, heart, brain, and spinal cord abnormalities in humans (*1*,*4*,*8**,**23*).

The potential economic effect of FZTs on alleviation of poverty is also a concern. Aquaculture in Vietnam is a major economic activity in rural areas. During 2000–2006, Vietnam tripled the value of its export of fish, increasing its revenue to >3 billion US dollars. Domestic availability of farmed fish is also a way of increasing protein availability to humans. Therefore, a newly recognized fish safety risk associated with aquaculture could have a serious constraint on market access (*24*), especially because consumer expectations and economic levels are increasing as predicted for Vietnam. These expectations can result in greater demand for safe fish by consumers, marketing agencies, and the tourist industry.

Our results showed a difference in infection rates of small trematodes between men (68.7%) and women (23.1%). Differences in liver infections with *C*. *sinensis* by sex of the patients are well known (*1*,*4*,*6*). Our results are similar to those of surveys conducted for this parasite in northern Vietnam (*25*,*26*). High infection rates for men in Vietnam are often associated with male-oriented social gatherings during which they consume raw or pickled fish, although this sex-related difference appears to be narrowing in some countries (*4*,*9*).

The relationship between prevalence and intensity of infection and age of the host is also a characteristic of FZT epidemiology (*1*,*4*). In our study, infection rates were higher for persons >40 years of age, a pattern believed to be caused by longer exposure and accumulation of parasites (*4*,*9*,*11*). However, the life span of intestinal flukes in humans is not well documented. Therefore, accumulation of worms as an explanation for age-related infection patterns is speculative. These behavioral factors in the epidemiology of FZT warrant greater collaboration between epidemiologists and anthropologists in designing approaches for mitigating risk in a population with great resistance to change in eating habits.
